# Learning curve estimation and insertion depth in Veress needle insertion using a conventional Veress needle and the VeressPlus™ needle

**DOI:** 10.1007/s00464-025-12273-4

**Published:** 2025-10-09

**Authors:** Tim Horeman-Franse, Roelf Postema, David Cefai, Sem Frederik Hardon, Predrag Andrejevic, Jenny Dankelman, Jean Calleja-Agius, Christian Camenzuli

**Affiliations:** 1 Department of Biomechanical Engineering, Delft University of Technology, Mekelweg 2, 2628 CD Delft, The Netherlands; 2 Department of Surgery, University Medical Centers Amsterdam, Location VUMC, De Boelelaan 1117, 1081 HV Amsterdam, The Netherlands; 3 Engineering Department, 2631CM, ProVinci Medtech, Nootdorp, The Netherlands; 4 Department of Surgery, Mater Dei Hospita, University of Malta, Msida, MSD2080 Malta; 5 Department of Anatomy, Faculty of Medicine and Surgery, University of Malta, Msida, MSD2080 Malta

**Keywords:** Laparoscopy, Entry technique, Veress needle, Safety mechanism

## Abstract

**Background:**

The Veress Needle (VN) is commonly used in establishing pneumoperitoneum in laparoscopic surgery. However, severe vascular and/or visceral complications can occur due to overshoot at the insertion of the VN in the abdominal cavity. In order to investigate whether the new VeressPLUS needle (VN+) could improve safety, the learning curve of this needle was compared to that of a conventional VN, under standardized conditions.

**Methods:**

In total, 26 residents and med students, without prior Veress needle experience, were recruited and randomly assigned to VN or the VN+ group. A learning curve plateau phase recognition model was developed and used to determine the learning curve of the participants who used either the VN or the VN+ needle on two Thiel-embalmed human cadavers. Insertion of the needles was done in a systematic way in the upper abdomen and insertion depth was measured under direct laparoscopic vision. At the end of the learning curve, the number of participants that reached a safe insertion depth between 5 and 15 mm was compared.

**Results:**

On average, it took the VN group 8 trials to reach and establish the plateau phase of the learning curve. The VN+ group showed no learning curve at all. At the 8th trial, a significant difference (*p* < 0.002) in average insertion depth was found in favor of the VN+ (mean: 5.4 mm SD 1.4) compared to the VN (mean: 12.7 mm SD 6). In the VN group and VN+ group, 46% versus 8% exceeded the safe insertion depth of 10 mm at the end of the learning curve.

**Conclusion:**

This study indicates that for novices, there is no learning curve for the VN+, when compared to VN. Moreover, in all cases, the insertion depths were significantly reduced (with more than 50%) while using the VN+ when compared to the VN.

**Supplementary Information:**

The online version contains supplementary material available at 10.1007/s00464-025-12273-4.

Laparoscopy has gained prominence due to its potential to minimize surgical wounds, thereby decreasing postoperative pain, accelerating recovery, and reducing the length of hospital stays. In order to be able to have adequate visualization and working space in the abdomen during laparoscopy, it is customary to create a pneumoperitoneum by insufflating a harmless gas, mostly CO_2_. Several methods can be used to establish this pneumoperitoneum, and one of the most frequently implemented involves a Veress Needle (VN). This special needle is utilized to inflate the abdomen with CO_2_ to create space for the safe introduction of the trocars that accommodate the laparoscopic camera and instruments. Named after the Hungarian physician Dr. János Veress who developed this innovative device around 1913, the VN has become the standard for many laparoscopic surgical specialties like gynecology, urology, and bariatric surgery [1]. Commonly used abdominal entry sites for the VN include the umbilicus or the left upper quadrant, also known as Palmer’s point [2]. The needle is inserted using a quick, controlled downward motion at the selected site while applying slight pressure to penetrate through the skin and underlying tissue. The VN features a spring-loaded mechanism that produces a distinct "click" when it enters the peritoneal cavity. The design of the commonly used VN incorporates a dual-function mechanism. It features a sharp (outer) trocar point that enables entry through the abdominal wall and into the peritoneal cavity, and inside of this sharp part of the needle is a blunt inner stylet which springs forward once resistance diminishes when the intra-abdominal cavity is reached. This blunt stylet is intended to prevent the sharp tip of the needle from puncturing the intra-abdominal organs and tissues. However, because the surgeon must force the needle through the abdominal wall tissues (subcutis, outer fascia, muscles, inner fascia, peritoneum), there is still a lot of kinetic force or momentum of the surgeons’ arm (and attached needle) in the small area of the tip of the needle when it enters the abdominal cavity. Consequently, due to the residual force through the very small area of the needle tip (although blunt), this could still lead to unintentional perforations of (hollow) organs (e.g., bowel, bladder, liver, spleen) or damage to vessels, leading to internal bleeding, perforation, or leakage of contents [3]. The chance of these complications occurring depends on the penetration or “insertion depth” in the abdomen (Fig. 1). When the needle is inserted further into the cavity than technically needed, this is called “Overshoot.” Therefore, it is important that the surgeon reacts quickly the moment the needle enters the abdominal cavity to reduce this overshoot to a minimum. Due to the aforementioned residual energy and the time delay in human reaction, there will, however, always be an amount of overshoot in clinical practice [4]. This can be reduced either by training surgeons to react as quickly as possible or by taking over control by electro-mechanical means like robot arms, end-stops, or force-release mechanisms [5, 6].

**Fig. 1:**
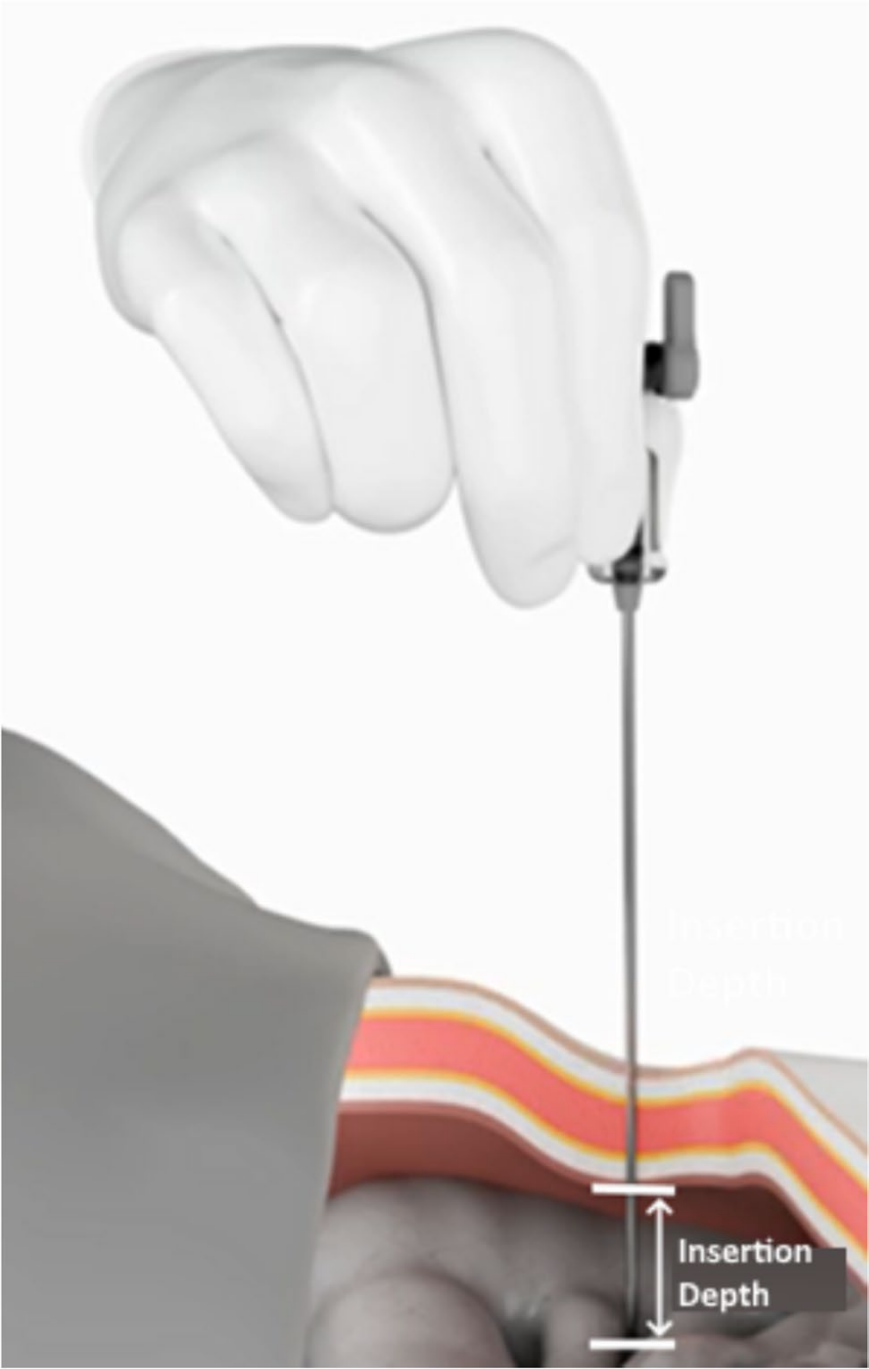
The insertion depth is defined as the insertion of the tip into the cavity after penetrating the layers of the abdominal wall. There is a direct relation between the insertion depth and the risk on undesired organ penetration that can lead to life-threatening complications

Using the VN effectively and safely requires a combination of knowledge, skill, and careful technique to minimize risk to the patient during laparoscopic surgery, and therefore, healthcare professionals should only perform this procedure after substantial training. The progression of technical skills training in laparoscopic surgery can be tracked by objectively monitoring so-called performance parameters [7, 8]. When trainees are asked to repeat standardized training tasks, these parameters can be used to plot learning curves (LCs) that indicate whether progression is still being made or that a plateau phase is reached, indicating proficiency with the task. In the case of VN insertion, no structured LC data could be found in the literature showing how much training is needed to master the use of safe VN handling. From a previous inventory study by our group conducted in collaboration with the European Association of Endoscopic Surgery [4], it became clear that there is no generally accepted number of insertions that need to be performed to reach a plateau phase. In fact, the opinion ranges from a couple of insertions on a phantom to hundreds of insertions on actual patients. From the same publication, it became clear that the safe insertion depth is considered to be between 0 and 10 mm by most of the respondents.

Therefore, the first aim of this study is to determine the learning curve (LC) related to the standard Veress needle insertion. As needle overshooting is seen as one of the main risks related to the use of the VN, a new type of VN, the VeressPlus™ (VN +) was developed with an integrated mechanical safety mechanism (Fig. 2). The detailed design of the functional parts of this safety mechanism can be found in our previous publication [5]. At the time of publication, this needle innovation is still in its experimental phase and not yet approved for human use.

**Fig. 2:**
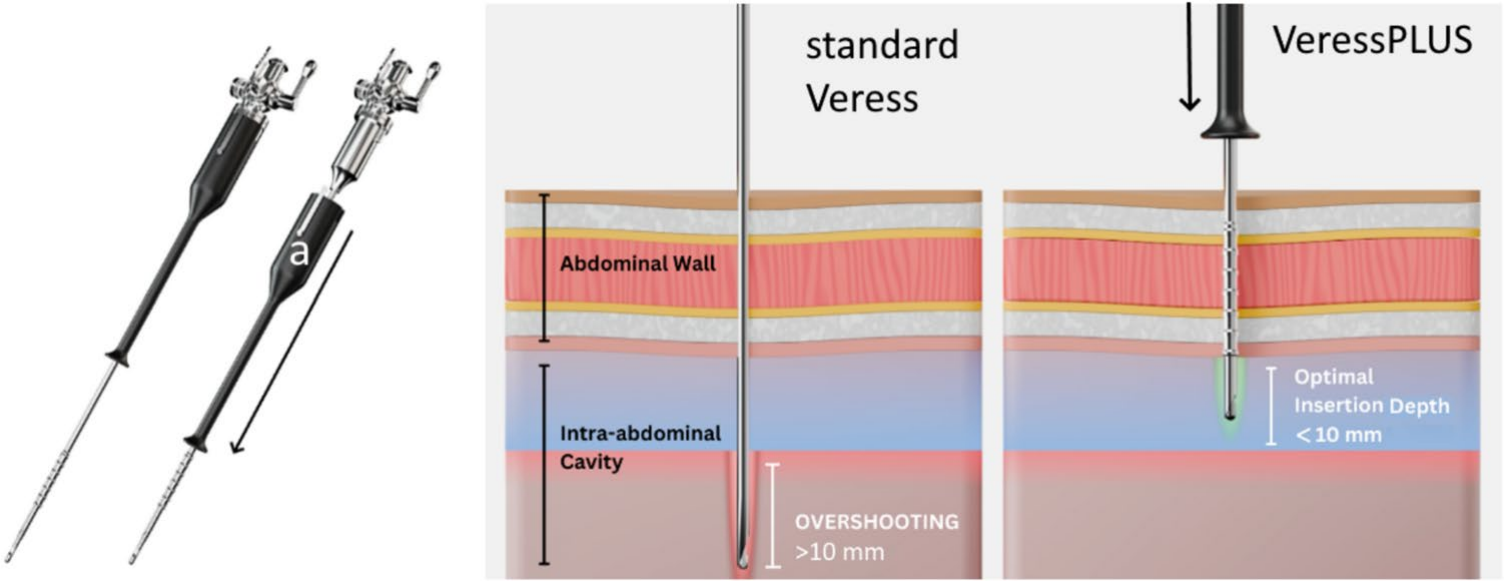
The VeressPLUS (VN+) needle. Left, the additional safety sheet (a) that is in contact with the hand can dislodge. Right, conventional Veress needle versus VeressPLUS shows that overshooting is prevented because the needle sheet dislodges after puncturing the peritoneum, leaving the needle and stylet in place at the Optimal Insertion Depth (OID) without driving it further into the cavity [5]

This mechanism immediately removes the driving force as soon as the tip enters the cavity, thereby reducing potential overshoot. This is made possible by a mechanism that disconnects the grip part (which has the surgeon’s fingers on it) from the VN at exactly the moment when the blunt inner stylet of the VN springs outwards. This is when all layers of the abdominal wall have been penetrated and the needle enters the abdominal cavity [5]. This immediate disconnection removes the kinetic force from the VN+ needle. In previous studies, we evaluated the VN+ in human cadavers, showing a significant reduction in overshoot of more than 50% [5, 6]. As it is still unclear how much time is needed for the trainees to master the skillset needed to use the VN+ safely, the second aim of this study is to compare the LC of the VN with the VN+.

## Methods

### Test setup

Two Thiel-embalmed human cadavers [9] were used to compare the overshoot between the new and conventional needle designs. Tissues of Thiel-embalmed bodies have good mechanical properties representative of living humans, which is relevant when testing instrument technology that relies on differences in stiffness, reaction force, and friction when interacting with tissues [10, 11].

In the first series of experiments with a VNc, the first body was an 86-year-old male who had died of colon cancer and Crohn’s disease (height: 170 cm; bodyweight: 75 kg, BMI = 26). The second body was a 79-year-old female (height: 160 cm; bodyweight: 86 kg, BMI = 33.6) who had died of a metastatic adenocarcinoma of the colon. In the second series of experiments conducted with the VN+ needle, the first body was an 86-year-old male who had died because of myocardial infarction (height: 170 cm; bodyweight: 75 kg, BMI = 26). The second body was a 79-year-old male who died of myocardial infarction (height: 179 cm; bodyweight: 87 kg, BMI = 27.2). Both bodies had a fully intact abdominal wall.

**Fig. 3:**
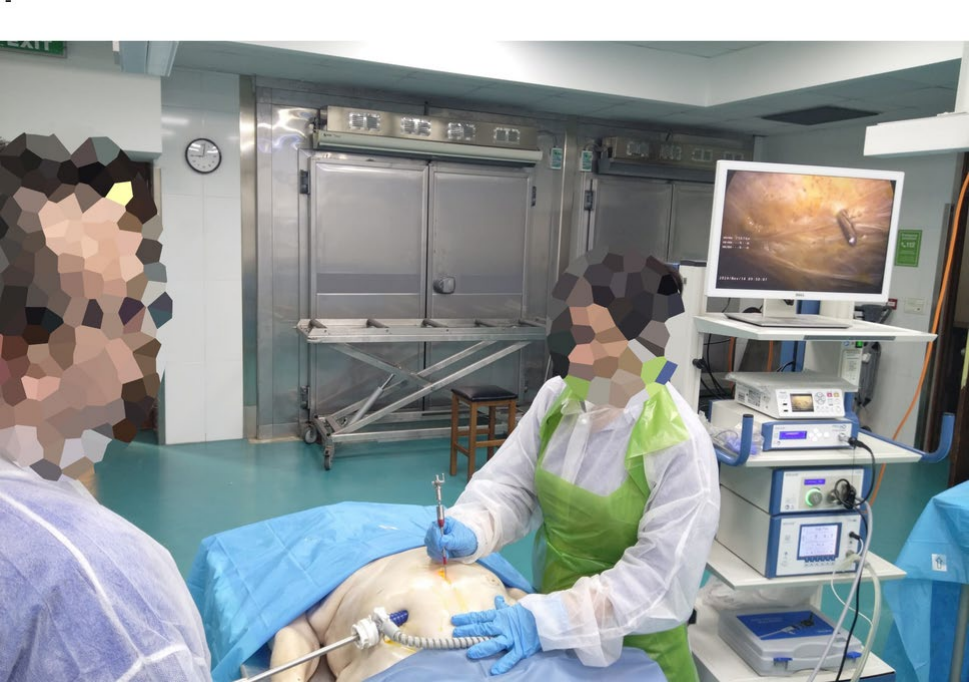
Setup used for both experiments. The research leader holds the scope while the participant places the needle on the indicated point (incisions made by the research leader)

The setup of the experiments can be seen in Fig. 3. A HD Aesculap laparoscopy tower setup (Aesculap, 3773 Corporate Parkway, Center Valley, 18034 PA, USA) was used with a 10 mm 30 degrees scope, which was inserted through a 12 mm trocar (Medtronic Netherlands, Larixplein 4, 5616VB Eindhoven, The Netherlands) in the abdomen just below the umbilicus. Intra-abdominal pressure was stabilized using CO_2_ to 4 mm Hg.

This pressure level was as low as possible to allow for unambiguous visualization of the insertions and almost equalling normal abdominal pressure. A commercial conventional VN (Trokamed GmbH, Geisingen, Germany) with a diameter of 2 mm and 145 mm length was used in this study as well as the proto-series VN+ needle [VeressPLUS, ProVinci Medtech, The Hague, the Netherlands], also with a diameter of 2 mm and 145 mm length (VN +). Video footage was used by three researchers to determine the insertion depth, indicated by the number of markings (which are 5 mm apart) on the part of the needle surface inside the abdomen. The study was registered under number FRECMDS-2021-134 of the Ethical Committee of the Faculty of Medicine and Surgery of the University of Malta.

### Protocol

Participants were included based on availability during the two measurement days, with a maximum of 32. This number was defined by the amount of available time slots of 30 min per participant. All participants were instructed about the study, were shown how the Veress mechanism works, and signed an informed consent form. During the instruction phase, they were allowed to see and feel both the VN and VN+ needles before actually using them. In order to minimize the risk of injuring organs upon entering the abdominal cavity, certain areas are dedicated for VN placement, such as Palmer’s point in the left upper quadrant of the abdomen [12]. In order to use the bodies as efficiently as possible, while offering a similar experience in terms of abdominal composition to all participants, the whole of the upper part of the abdomen was used for the insertions from cranial to caudal to ensure that an abdominal wall part without defect or lesion was used for each attempt. During the VN placements and measurements, the participants were standing with their backs to the video monitor, so they were blinded to what was happening inside the abdomen. Within the first series of experiments, the participants used the VN needle on two embalmed bodies. During the second series, the VN+ needle was used on the other two bodies. Insertion of the VN+ was considered successful when the safety mechanism became fully unlocked. If not, the attempt was noted as an unsuccessful trial. Participants were free to choose how they used the fingers of the hand holding the needle or the other hand for support or stabilization of the needle. During the execution part of the session, no feedback was provided that could influence performance. The participants were divided in alternating order over the groups based on arrival.

### Real-time Plateau phase recognition

**Fig. 4:**
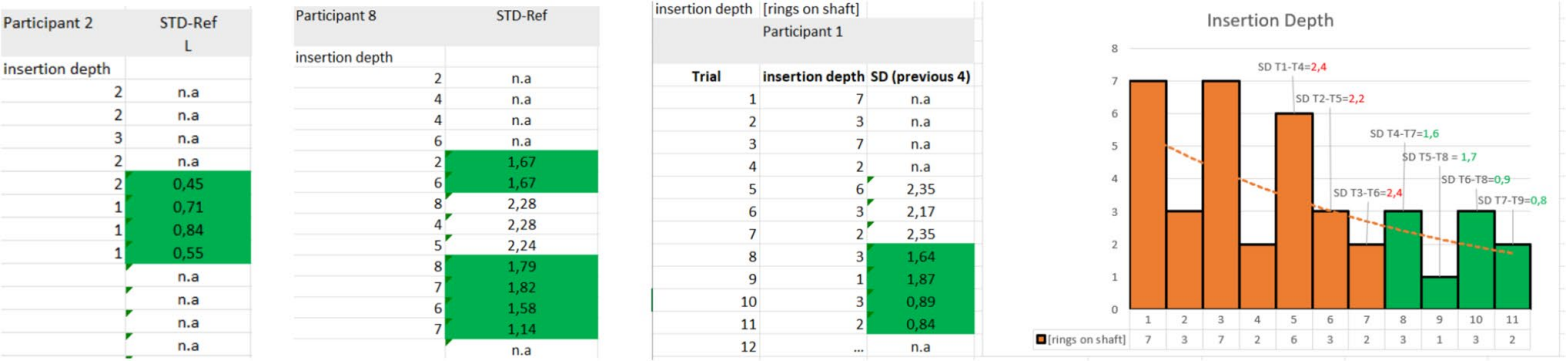
Example of 3 typical results from a simple real-time predictive model constructed in excel (Supplemental File 2) showed the researcher when the insertion sequence should be stopped. Left example, the first 4 measurements were consistent enough to indicate a plateau phase. Middle-left example, measurements are not consistent enough and still show progression till the 9th measurement. Middle Right example, after 8 measurements, a consistent plateau phase is reached as shown in the corresponding LC representation in the Right figure

The number of insertions per participant determined if and when participants entered the plateau phase and thus mastered the needle mechanism. Therefore, a real-time predictive model was constructed in Excel (Supplemental File 1) that showed the researcher when the insertion sequence could be stopped. For every 4 previous measurements, the Standard Deviation (SD) was calculated. As soon as this SD was less than 10 mm (2 indicator markings), the cell turned green. After the first green cell, the participant was asked to continue inserting the needle till 4 executive cells in a row were green (Fig. 4—left). If a following cell turned white again, the participant needed to continue till obtaining a following block of 4 green cells in a row (Fig. 4—middle left).

### Data comparison and statistical analysis

A potential learning curve was identified based on a linear mixed-effects model to account for repeated measures nested within participants. This approach models both fixed effects (e.g., trial number) and random effects (e.g., participant-specific intercepts), allowing for inter-individual variability, and a *p* value < 0.05 was considered a significant difference (Supplemental File 2). In addition, any differences in maximum insertion depth between the VN and the VN+ groups were determined using a Student’s t-test (SPSS v16, SPSS, Inc., Chicago, IL) at the end of the LC. A *p* value < 0.05 was considered a significant difference. At the end of the learning curve, the number of participants that reached an insertion depth below 10 mm was compared. This optimal insertion depth zone between 0 and 10 mm was extracted from a previous inventory study executed among EAES members (Fig. 2B) [4].

## Results

### VN measurement series

**Fig. 5:**
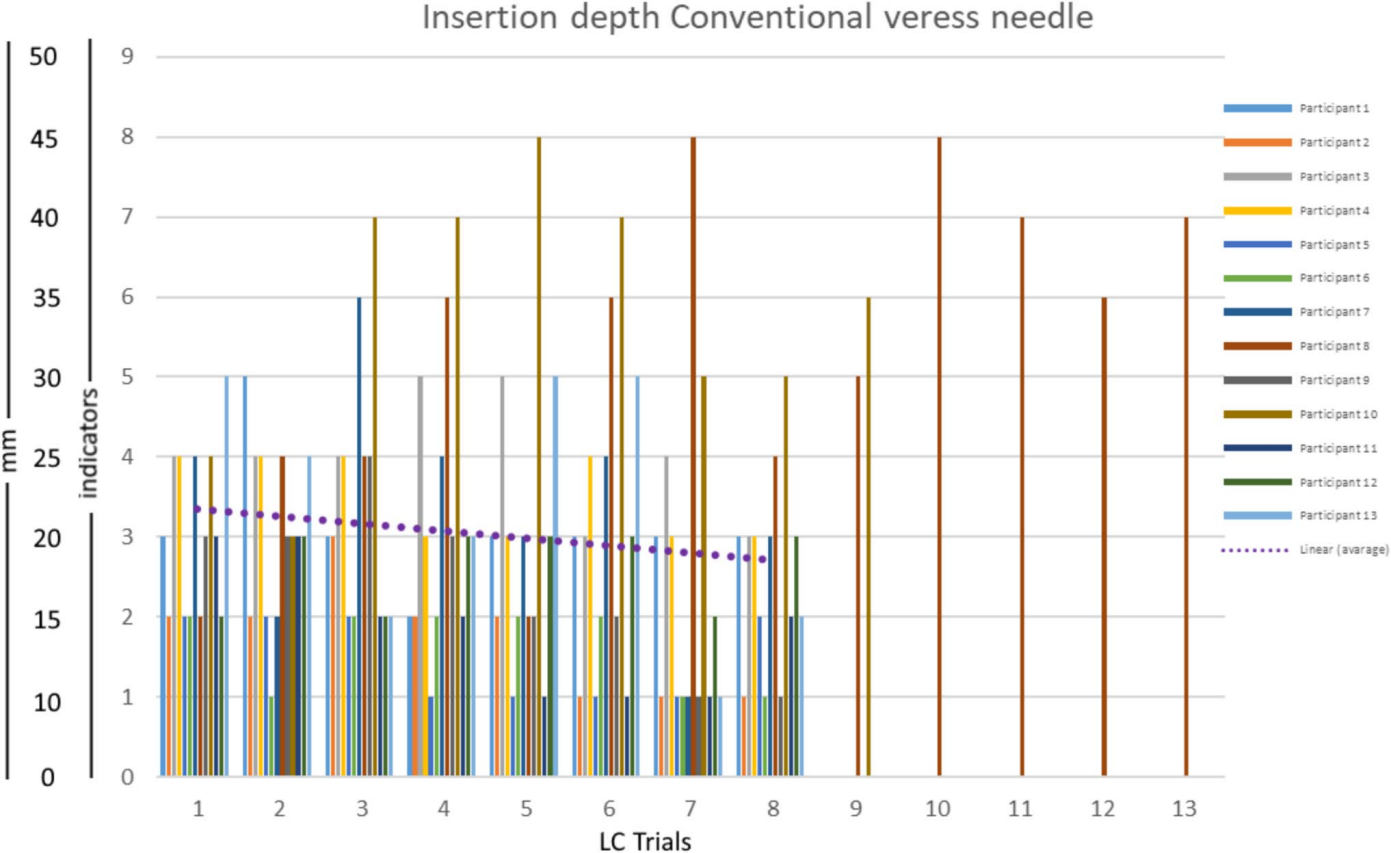
The insertion data over time (trials) of the 13 participants that worked with the Conventional Veress needle. Only two participants did not reach the plateau phase in the first 8 trials

The total of 26 participants were equally divided into 2 groups, one group using the VN and the other group using the VN+. In the VN measurement series, eight out of 13 participants were female, of whom only one was left-handed. In the VN+ measurement series, six of the 13 participants were female, and all were right-handed. All 13 participants included in this study were classified as novice, which was defined as having had no previous experience with using or handling a VN. Figure 5 shows the insertion depth over time with the LC indication of the measurement Series 1 with the VN. Six participants in this group needed a maximum of 8 repetitions before arriving at 4 consecutive insertions with a data variation of less than SD = 2. Two individuals stabilized at very high insertion depths above 25 mm at the end of the LC. Especially, those two participants were advised to train more with the Veress needle before using it on live patients.

### VN+ measurement series

**Fig. 6:**
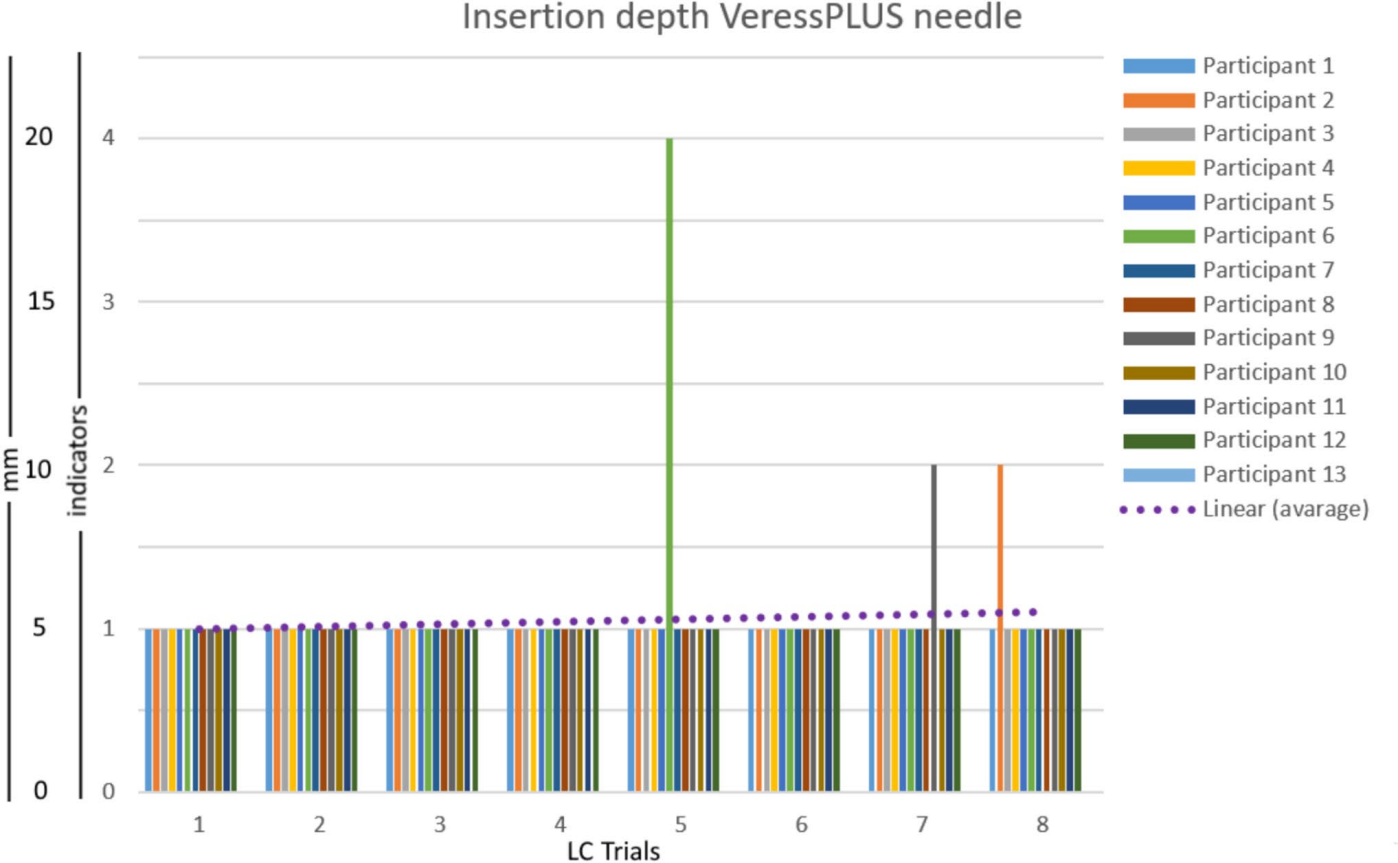
The insertion data over time (trials) of the 13 participants that worked with the VN+. All participants reached the plateau phase in the first 8 trials

Figure 6 shows the insertion depth over time with LC indication of Series 2, which was performed with the VN+. All 13 participants included in this study were classified as novice. From these 13, only two participants showed more data variation than SD = 2 after the 4th trial and had to insert the needle more than 8 times before hitting 4 repeated insertions, with an insertion data variation of less than SD = 2. During Series 2, the VN+ mechanism dislodged 4 times during insertion through the abdominal wall, all in the first attempt of 4 different participants. It was observed that pulling the needle backwards for repositioning during insertion (against the protocol) resulted in dislodgement of the hand grip due to a loss of actuation force. When the system dislodged, a re-attempt was allowed, and the instruction was repeated. All participants in this group needed a maximum of 8 repetitions before arriving at 4 consecutive insertions with a data variation of less than SD = 2. It was also observed that, from all participants in this group, two individuals were responsible for the three larger insertion depths. The larger insertion depth of 4 mark (20 mm) resulted from direct contact between the peritoneum of the abdominal wall and the underlying organs.

### Data comparison and statistical analysis

Supplemental File 1 provides the data obtained from the experiments. Comparing the VN and VN+ data at the 8th trial, a significant difference of 7.3 mm (p < 0.002) was found between the total averaged insertion depth for VN of 12.7 SD5.9 mm and VN+ of 5.4 SD1.4 mm. A comparison of all eight trials shows a significant difference (p < 0.001) between the total averaged insertion depth for the VN+ of 5.3 SD0.4 mm and the VN of 15.1 SD1.8 mm. In the VN group and VN+ group, 46% versus 8% exceeded the safe insertion depth at the end of the experiment. Regarding the regression analysis (Supplemental File 2), the VN group showed a significant negative effect of trial number on insertion depth: regression coefficient (*β*) − 0.111 (*p* = 0.015), indicating a typical learning curve. The significant random intercept (*p* = 0.026) reflects the inter-individual variation in this group. The VN+ group showed no significant learning effect: regression coefficient (β) + 0.014 (*p* = 0.323), and between-subject variance was negligible.

## Discussion

The LC data presented in this study help to put the results obtained from the EAES Veress needle inventory study [4] in perspective as qualitative data obtained in a standardized realistic setting is difficult to obtain due to the related complexity and costs of this kind of studies [13]. Despite more than 46% of the participants that used a VN did not reach the optimal insertion depth of 10 mm, the group average showed a learning curve throughout the trials of the experiment. This learning curve was absent for the group or individuals who worked with the VN+ safety mechanism. The large inter-human variation, which was found in the series with the VN, indicated that surgical residents do not obtain the required skillset in a similar way. It was especially interesting to see that two participants deviated largely from the group performance and were not able to achieve comparable insertion levels to the rest of the VN group participants at the end of the plateau phase. Moreover, 67% of the participants who used the VN at the 8th trial were not able to reach the optimal insertion depth of 10 mm, while in the VN+ group all participants reached the optimal insertion depth of 10 mm at the end of the LC trials. In fact, all but one managed to limit their insertion depth to only 5 mm showing the potential of the additional safety mechanism.

Most participants reacted surprised and positive to working with the VN+ concept. In the post-session discussion they indicated that they had no idea that a mechanical system that relies on a force equilibrium could work independent of the thickness of the layers and insertion method, as most needle-like devices that have an insertion limiter integrated rely on a hard stop that comes in contact with tissue and prevents further progression of the sharp element. Despite the low error rate, the observed dislodgement indicated that proper hands-on instructions and a prior hands-on training trial were needed for the participants before the system mechanics (of the VN +) could be fully understood. In this instruction, it is important to explain to the user why only the grip should be touched and not the other components (e.g., crane, coupling parts or needle) and to apply the needle force in a constant fashion toward the incision.

Although the focus during this experiment was on the determination of LCs of the VN insertion, the absolute insertion values are comparable with the results from our previous experiments on Thiel-embalmed bodies [6]. However, the data also indicate that participants need to learn how to hold the VN+ in a proper way for the mechanism to fully dislodge, as the first attempt sometimes went wrong. The absence of a learning effect indicates that when the system is operated properly, the insertion depth is determined by the VN+ safety mechanism and not by the gained experience.

It was observed in both experiments that when the needle did penetrate through the peritoneum and the inner stylet consequently did not spring out immediately, there was direct contact between the peritoneum and the underlying organs. When using the VN, it became evident that for the Veress mechanism to work properly, two factors are really important. First, the needle needs to be really sharp to prevent abdominal wall layers from being pushed into the abdominal cavity instead of having a clean cut through each layer. Especially when the insertion point is further away from the ribs, it can be argued that a blunt needle tip more likely presses the tissues further down than needed, resulting in contact between the peritoneum and organs which could lead to complications. Secondly, the needle and blunt stylet need to be really clean from the inside. As soon as there is a large build-up of dirt or a biofilm, between both tubular parts of the needle, the debris acts as a viscosity damper when the blunt stylet moves inside the needle when it is supposed to shout out for protection after penetrating through the peritoneum. As the decoupling mechanism of the VN+ mechanism relies on this action, the dislodgement is also delayed, resulting in a deeper needle insertion.

### Study limitations

Only two bodies with a relatively normal posture were used for the experiments in this study. As the tissue layer thickness of the abdominal wall of obese patients can be different from patients with a normal BMI, the influence on the VN+ functioning and ease of positioning within the learning curve should be investigated in bodies with different BMIs in future studies, including registration of the abdominal wall thickness. For the trial length calculation, a standard deviation of 2 indicators (10 mm) over the last 4 trials was considered to be a plateau phase and an indication that the experiment could end. Most likely, the detectable LC becomes longer than eight trials when the measurements become more accurate and the novices further improve their insertion skills toward an expert level. In further research, this can be done by adding more indicator marks (e.g., finer scale) on the needle and using a threshold SD shorter than two. Finally, it should be noted that all participants in this trial were novices, and the performance may differ among experts. To reflect actual performance across a broader patient population, a more robust design would include attempts on different cadavers as well as cadavers of varying body habitus, thereby allowing assessment of both intra- and inter-cadaver variability and providing a more accurate representation of clinical practice.

## Conclusion

The Veress needle learning curve study showed that most, but not all participants are able to reach a LC plateau phase within 8 trials. With the added VN+ dislodgment mechanism, all participants are able to place the needle with a much lower insertion depth in a much more consistent way from the start of the session. While the Veress needle in any form or shape is a valuable tool for laparoscopic surgery, awareness of potential complications related to practice or technical components is crucial for safe use. Proper training, technique, and patient monitoring can help minimize these risks. Despite the great results, a larger multicentre field study, executed by an independent team, is needed to truly investigate the potential of this new working principle when integrated in the Veress needle.

## Supplementary Information

Below is the link to the electronic supplementary material.

Supplementary file1 (XLSX 69 KB)

Supplementary file2 (PDF 108 KB)
