# Supplementary material for: Learning curve estimation and insertion depth in Veress needle insertion using a conventional Veress needle and the VeressPlus™ needle
Source: Surg Endosc. 2025 Oct 9;40(1):247–54. doi: 10.1007/s00464-025-12273-4 (PMC12823718; doi:10.1007/s00464-025-12273-4)
Supplement: Supplementary file 2 — Supplementary file2 (PDF 108 KB) [file 464_2025_12273_MOESM2_ESM.pdf]

Mixed Model Output Summary: VNc and VN+

VNc Results

Descriptive Statistics

Participant_ID		Count	Mean	Standard Deviation	Coefficient of Variation
P01	Insertion_Depth_mm	8	3,13	,835	26,7%
	Trial_ID	8	4,50	2,449	54,4%
P02	Insertion_Depth_mm	8	1,75	,707	40,4%
	Trial_ID	8	4,50	2,449	54,4%
P03	Insertion_Depth_mm	8	4,00	,756	18,9%
	Trial_ID	8	4,50	2,449	54,4%
P04	Insertion_Depth_mm	8	3,50	,535	15,3%
	Trial_ID	8	4,50	2,449	54,4%
P05	Insertion_Depth_mm	8	1,50	,535	35,6%
	Trial_ID	8	4,50	2,449	54,4%
P06	Insertion_Depth_mm	8	1,63	,518	31,8%
	Trial_ID	8	4,50	2,449	54,4%
P07	Insertion_Depth_mm	8	3,38	1,506	44,6%
	Trial_ID	8	4,50	2,449	54,4%
P08	Insertion_Depth_mm	8	4,00	1,512	37,8%
	Trial_ID	8	4,50	2,449	54,4%
P09	Insertion_Depth_mm	8	2,38	1,061	44,7%
	Trial_ID	8	4,50	2,449	54,4%
P10	Insertion_Depth_mm	8	5,75	1,753	30,5%
	Trial_ID	8	4,50	2,449	54,4%
P11	Insertion_Depth_mm	8	1,88	,835	44,5%
	Trial_ID	8	4,50	2,449	54,4%
P12	Insertion_Depth_mm	8	2,63	,518	19,7%
	Trial_ID	8	4,50	2,449	54,4%
P13	Insertion_Depth_mm	8	3,38	1,598	47,3%
	Trial_ID	8	4,50	2,449	54,4%

**Total**	**Insertion_Depth_mm**	**104**	**2,99**	**1,542**	**51,6%**
	**Trial_ID**	**104**	**4,50**	**2,302**	**51,2%**

Estimates of Fixed Effects[a]

Parameter	Estimate	Std. Error	df	t	Sig.	95% Confidence Interval Lower Bound	95% Confidence Interval Upper Bound
**Intercept**	**3,489**	**,391**	**21,689**	**8,919**	**<,001**	**2,677**	**4,301**
**Trial_ID**	**-,111**	**,045**	**90,000**	**-2,489**	**,015**	**-,199**	**-,022**

a. Dependent Variable: Insertion_Depth_mm.

Estimates of Covariance Parameters[a]

Parameter		Estimate	Std. Error	Wald Z	Sig.	95% Confidence Interval Lower Bound	95% Confidence Interval Upper Bound
**Residual**		**1,082**	**,161**	**6,708**	**<,001**	**,808**	**1,450**
**Intercept [subject = Participant_ID]**	**Variance**	**1,332**	**,599**	**2,222**	**,026**	**,551**	**3,218**

a. Dependent Variable: Insertion_Depth_mm.

VN+ Results

Descriptive Statistics

Participant_ID		Count	Mean	Standard Deviation	Coefficient of Variation
P01	Insertion_Depth_mm	8	1,00	,000	0,0%
	Trial_ID	8	4,50	2,449	54,4%
P02	Insertion_Depth_mm	8	1,13	,354	31,4%
	Trial_ID	8	4,50	2,449	54,4%
P03	Insertion_Depth_mm	8	1,00	,000	0,0%
	Trial_ID	8	4,50	2,449	54,4%
P04	Insertion_Depth_mm	8	1,00	,000	0,0%
	Trial_ID	8	4,50	2,449	54,4%
P05	Insertion_Depth_mm	8	1,00	,000	0,0%
	Trial_ID	8	4,50	2,449	54,4%
P06	Insertion_Depth_mm	8	1,38	1,061	77,1%
	Trial_ID	8	4,50	2,449	54,4%
P07	Insertion_Depth_mm	8	1,00	,000	0,0%
	Trial_ID	8	4,50	2,449	54,4%
P08	Insertion_Depth_mm	8	1,00	,000	0,0%
	Trial_ID	8	4,50	2,449	54,4%
P09	Insertion_Depth_mm	8	1,13	,354	31,4%
	Trial_ID	8	4,50	2,449	54,4%
P10	Insertion_Depth_mm	8	1,00	,000	0,0%
	Trial_ID	8	4,50	2,449	54,4%
P11	Insertion_Depth_mm	8	1,00	,000	0,0%
	Trial_ID	8	4,50	2,449	54,4%
P12	Insertion_Depth_mm	8	1,00	,000	0,0%
	Trial_ID	8	4,50	2,449	54,4%
P13	Insertion_Depth_mm	8	1,00	,000	0,0%
	Trial_ID	8	4,50	2,449	54,4%
Total	Insertion_Depth_mm	104	1,05	,323	30,8%

Trial_ID	104	4,50	2,302	51,2%

Estimates of Fixed Effects[a]

						95% Confidence Interval	
Parameter	Estimate	Std. Error	df	t	Sig.	Lower Bound	Upper Bound
Intercept	,986	,070	102	14,119	<,001	,848	1,125
Trial_ID	,014	,014	102	,993	,323	-,014	,041

a. Dependent Variable: Insertion_Depth_mm.

Estimates of Covariance Parameters[a]

						95% Confidence Interval	
Parameter		Estimate	Std. Error	Wald Z	Sig.	Lower Bound	Upper Bound
Residual		,104	,015	7,141	<,001	,079	,137
Intercept [subject = Participant_ID]	Variance	,000[b]	,000	.	.	.	.

a. Dependent Variable: Insertion_Depth_mm.

b. This covariance parameter is redundant. The test statistic and confidence interval cannot be computed.
